# Effect of Tripropylene Glycol Diacrylate Doping on the Uniformity of Phenanthrenequinone/Poly(Methyl Methacrylate) Photopolymer

**DOI:** 10.3390/polym18151851

**Published:** 2026-07-28

**Authors:** Enqiang Wu, Junhui Wu, Shenghui Ke, Jianlei Li, Erkang Yang, Xuelin Wang, Jun Xie, Jianwei Wu, Xiaodi Tan

**Affiliations:** 1 College of Photonic and Electronic Engineering, Fujian Normal University, Fuzhou 350117, Chinawy1742537418@163.com (J.W.); 2 Information Photonics Research Center, Key Laboratory of Optoelectronic Science and for Medicine of Ministry of Education, Fujian Provincial Key Laboratory of Photonics Technology, Fujian Provincial Engineering Technology Research Center of Photoelectric Sensing Application, Fujian Normal University, Fuzhou 350117, China

**Keywords:** holographic storage, TPGDA, photopolymer, performance uniformity

## Abstract

To address the key issues of uneven distribution of functional groups, large dispersion of holographic storage performance across different regions, and poor consistency of storage capacity in traditional PQ/PMMA holographic storage photopolymers, this paper introduces a low-viscosity reactive diluent, tripropylene glycol diacrylate (TPGDA), to modify the matrix. Leveraging the viscosity-reducing and double-bond crosslinking properties of TPGDA, the molecular diffusion behavior of the system was regulated. The effects of TPGDA doping ratio, the ratio of photosensitizer PQ to thermal initiator AIBN, and post-curing process on the holographic performance uniformity of the material were systematically investigated. The uniformity was quantitatively evaluated by the variance of diffraction efficiency at different points. Visible light absorption spectra and Fourier-transform infrared (FT-IR) spectroscopy were employed to reveal the modification mechanism from the perspective of functional group distribution. Actual-data read/write tests were conducted using a collinear holographic storage system. The experimental results show that the optimal TPGDA doping concentration is 40 wt%. For the optimized formulation TPGDA:MMA:AIBN:PQ = 8 g:12 g:0.20 g:0.18 g, the modified material achieves an average diffraction efficiency of 76.58%, and the diffraction efficiency variance decreases from 23.49 (pristine matrix) to 1.64, indicating a significant improvement in performance uniformity. Compared with pure PQ/PMMA, the modified material exhibits an approximately 1.95-fold increase in maximum diffraction efficiency, a 2-fold increase in recording rate, and a 1.6–1.75-fold increase in refractive index modulation. FT-IR spectroscopy confirms that TPGDA optimizes the spatial distribution uniformity of C=C and C=O functional groups. In collinear holographic measurements, the bit error rate (*BER*) variance of the modified sample is reduced by 40% relative to the pristine matrix, achieving homogeneous storage performance across the entire area while maintaining comparable signal-to-noise ratio (*SNR*) and *BER*. Additional short-time UV post-curing can further enhance the diffraction efficiency and refractive index modulation, and a thinner substrate can avoid performance fluctuations caused by incomplete thermal curing of thick samples. This study achieves directional optimization of the holographic uniformity of PQ/PMMA through reactive diluent viscosity reduction modification, providing a new strategy for the formulation design and engineering preparation of high-consistency holographic storage photopolymers.

## 1. Introduction

In the current era of big data, the global data volume is increasing exponentially and is expected to reach 527.47 ZB by 2029. Conventional data storage methods suffer from limitations such as high cost and short storage lifespan, making it difficult to meet the storage demands of the era. In 1948, the concept of holography was first proposed by Dennis Gabor [1]. Since then, holographic technology has been widely applied in many fields, including three-dimensional imaging [2,3,4,5], information encryption and security [6,7,8,9,10], and holographic data storage [11,12,13]. Among them, holographic data storage technology, owing to the selective multiplexing property imparted by the Bragg effect, has shown great potential in the field of data storage and is therefore widely regarded by the industry as a promising candidate for future mainstream storage technologies [14,15].

Current research on holographic storage mainly focuses on three types of materials: photorefractive materials, photochromic materials, and photopolymers [16]. Among photopolymers, phenanthrenequinone-doped poly(methyl methacrylate) (PQ/PMMA) photopolymer, which uses PMMA as the matrix and PQ as the photosensitizer, has become a highly promising recording medium for holographic storage technology due to its high photosensitivity, excellent structural stability, and negligible light-induced volume shrinkage. This material also offers advantages such as simple preparation, low cost, and high-resolution data storage capability [17,18]. These features have attracted extensive research interest worldwide. To improve the material properties of PQ/PMMA, various modification strategies have been proposed, ranging from the initial development and analysis by Veniaminov et al. [19] to the various strategies proposed by later researchers, such as doping with other comonomers (e.g., the incorporation of acrylamide, AA, which improved the heat resistance from 100 °C to 200 °C) [20], adding nanoparticles (e.g., the introduction of silver nanoparticles shortened the response time by 67% and increased the diffraction efficiency by 18.5%, while the addition of fullerenes enhanced the diffraction efficiency by 51%) [21,22], and introducing co-photoinitiators (e.g., triethanolamine, TEA, which increased the photosensitivity by tenfold) [23], etc. Through these methods, certain aspects such as diffraction efficiency, photosensitivity, photopolymerization shrinkage, refractive index modulation, and information recording capability can be improved, but a comprehensive enhancement is not achieved. Moreover, few of these studies have investigated the performance uniformity of PQ/PMMA. However, in practical holographic storage applications, the storage effect varies considerably across different regions of the same material, leading to differences in storage capacity at different locations.

In the PQ/PMMA system, the concentration of C=C bonds is a key factor determining material performance. Lin et al. [24,25] analyzed the photoreaction process during PQ/PMMA recording in 2003. Their work showed that the C=O bond of PQ reacts with the C=C bond of MMA to form photoproducts. Using FT-IR, nuclear magnetic resonance (NMR) spectroscopy, and gel permeation chromatography (GPC), they identified the structures of the photoproducts in the PQ/PMMA material. The experimental results demonstrated that PQ can react with both PMMA and MMA to form photoproducts PQ/PMMA and PQ/MMA. Therefore, the storage performance at different locations of PQ/PMMA is mainly influenced by the C=C and C=O bonds, and the distribution uniformity of these two functional groups is governed by molecular diffusion and reaction kinetics, in which the system viscosity plays a crucial role.

Many experiments have addressed the relationship between viscosity and molecular motion. Generally, it is known that an increase in solution viscosity hinders molecular motion [26]. This theory has been confirmed by numerous subsequent studies [27,28,29,30,31,32]. Tripropylene glycol diacrylate (TPGDA) can not only efficiently reduce the system viscosity of high-viscosity resins with its extremely low intrinsic viscosity [33,34,35,36,37] and improve processing leveling, but more importantly, its two terminal acrylate double bonds can participate in UV-curing crosslinking reactions [38] and become part of the final network, avoiding problems associated with solvent evaporation. This allows researchers to directly adjust the flexibility, adhesion, and internal stress of the cured film through the amount of TPGDA used. From a physical property perspective, TPGDA itself is a low-viscosity, high-boiling-point liquid. Its typical viscosity is about 25 mPa·s at 25 °C, which enables it to efficiently “dilute” or “reduce the viscosity” of high-viscosity base resins. Simple physical blending can significantly improve the fluidity, sprayability, or blade coating properties of the resin system, which is crucial for meeting the requirements of practical production processes such as coating and printing [33]. As a viscosity diluent, TPGDA can reduce the material viscosity, allowing molecules to move more freely. The interference recording process of PQ/PMMA mainly involves the reaction of the C=O bond of PQ with the C=C bond of PMMA under illumination; the extent of this reaction determines the final recording performance. By introducing TPGDA into PQ/PMMA, molecular motion is promoted, leading to a more uniform distribution of C=C and C=O bonds, thereby improving the performance uniformity of the material and solving the problem of varying storage capacity across different regions of the material during holographic storage. Furthermore, the TPGDA used in this study contains two C=C bonds, which allows better regulation of material performance [39].

This study addresses the key problems of uneven distribution of functional groups and large dispersion of holographic performance in different regions of conventional PQ/PMMA materials by introducing a low-viscosity reactive diluent TPGDA to modify the matrix, exploiting its viscosity-reducing and double-bond crosslinking properties to control the molecular diffusion behavior of the system. First, the effect of TPGDA doping ratio on the diffraction efficiency of the material was systematically investigated, and the variance of the maximum diffraction efficiency was calculated, determining 40 wt% as the optimal doping ratio. At this concentration, the performance uniformity of the modified material was significantly better than that of the undoped sample, and the maximum diffraction efficiency increased by approximately 20%. On this basis, the ratio of photosensitizer PQ to thermal initiator AIBN was further optimized, and the optimal formulation was determined to be TPGDA:MMA:AIBN:PQ = 8 g:12 g:0.20 g:0.18 g. To address the problem of incomplete thermal curing of thick samples after TPGDA introduction, the effects of a short-time UV post-curing process (100 s) and sample thickness (1.0 mm and 0.5 mm) on the stability of diffraction efficiency were investigated. FT-IR spectroscopy was used to perform variance analysis on the absorbance ratio of C=C to C=O bonds in different regions of the samples, revealing the mechanism by which TPGDA improves performance uniformity from the perspective of microscopic functional group distribution. Finally, actual-data read/write tests were carried out on the optimized TPGDA-PQ/PMMA using a collinear holographic storage system, and the bit error rate (*BER*) and signal-to-noise ratio (*SNR*) under different recording times were measured. The preliminary experimental results show that the introduction of TPGDA increases the recording speed by approximately 2-fold, and without significantly sacrificing the average *BER* and *SNR*, the *BER* variance is significantly reduced compared with that of the undoped sample, confirming that TPGDA doping enhances the storage performance uniformity of the material. This study achieves directional optimization of the holographic uniformity of PQ/PMMA through reactive diluent viscosity reduction modification, providing a new approach for the formulation design and engineering preparation of high-consistency holographic storage photopolymers, and offers important reference value for promoting the practical application of holographic storage technology.

## 2. Materials and Methods

In this paper, the photopolymer PQ/PMMA material and the TPGDA-doped material were prepared by thermal polymerization [40,41,42]. The main components of the PQ/PMMA polymer material include a monomer, thermal initiator, and photosensitizer. The monomer, methyl methacrylate (MMA), is a colorless transparent liquid, volatile and slightly soluble in water, and can self-polymerize to form poly(methyl methacrylate) (PMMA). The thermal initiator, azobisisobutyronitrile (AIBN), is a white crystalline powder, insoluble in water, easily decomposes upon heating, and promotes the polymerization of MMA into PMMA. The photosensitizer, phenanthrenequinone (PQ), is an orange-yellow needle-like crystalline powder, insoluble in water. The doped TPGDA is a reactive diluent, a colorless transparent liquid, insoluble in water. Detailed information on the above reagents is listed in Table 1.

**Figure 1:**
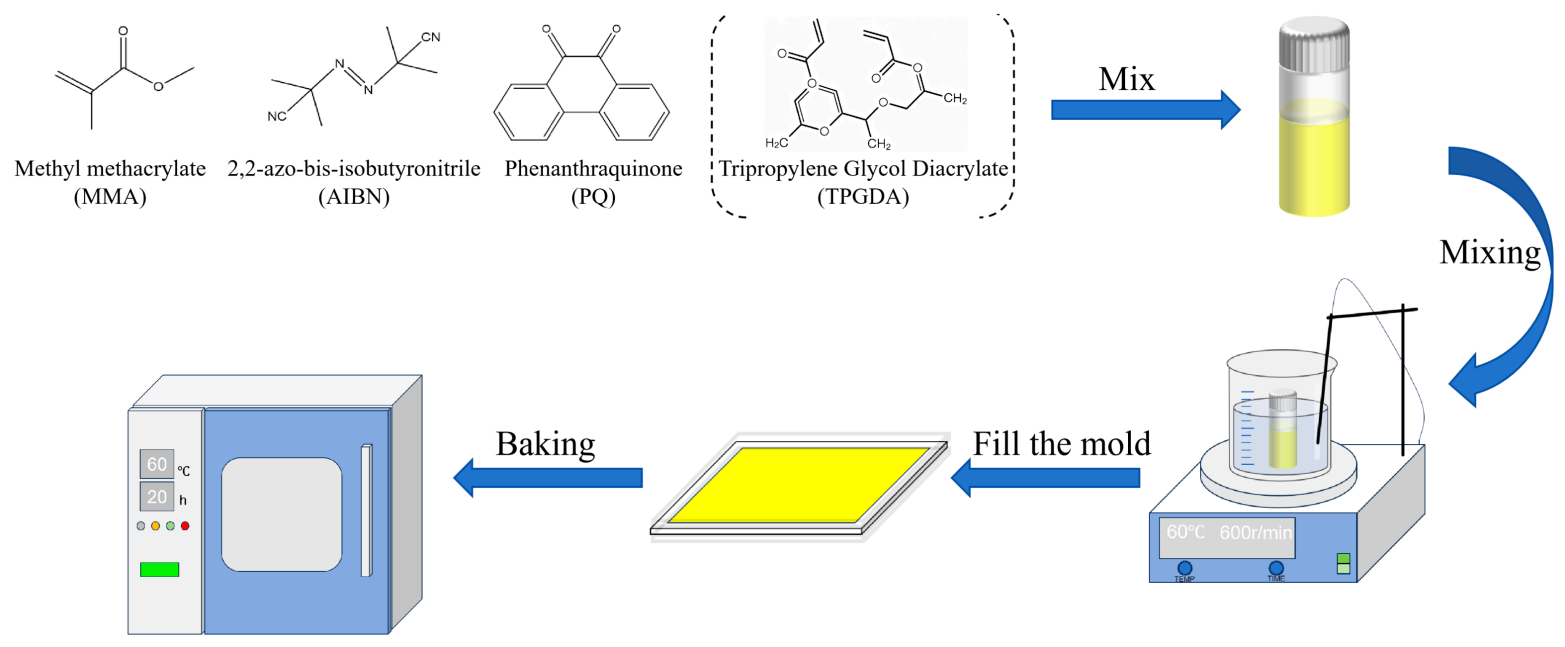
Sample preparation process.

The entire process of material preparation was carried out while avoiding exposure to ambient light with a wavelength below 550 nm. The specific preparation steps are illustrated in Figure 1. First, pre-experimental preparations: Turn on the ultrasonic oscillator, constant-temperature magnetic stirrer, and oven, all heated to 60 °C. Prepare the glass bottles, glass slides, polytetrafluoroethylene (PTFE) spacers, and stirring bars needed for the experiment. Next, weigh the chemicals according to the specified ratios. The conventional formulation of PQ/PMMA is MMA:AIBN:PQ = 100:1:1 [43]. In this study, 0.2 g each of AIBN and PQ were used. The weighing errors for MMA, AIBN, and PQ were ±0.0005 g, and the weighing accuracy for TPGDA was ±0.0100 g. The proportions of the samples prepared in this experiment are shown in Table 2. To ensure uniform mixing of the components after weighing, the weighed samples were placed in an ultrasonic cleaner at 60 °C for ultrasonic oscillation for 15 min. After oscillation, the samples were placed in a constant-temperature magnetic stirrer and stirred at 60 °C and 600 r/min. Undoped materials were stirred for 60 min, while for TPGDA-doped materials, since TPGDA can act as a crosslinking agent and excessive stirring would cause rapid polymerization and explosion of MMA, the stirring was stopped when the vortex was about to disappear. Finally, the well-stirred solution was poured into PTFE molds with a thickness of 1 mm, clamped with glass slides, and placed in an oven for thermal curing. For the collinear holographic system, reflective PQ/PMMA samples with a thickness of 0.5 mm were required and were cured at 50 °C for 12 h. All other materials in this study were 1 mm thick and were cured at 60 °C for 20 h. All prepared samples were stored in aluminum foil bags away from light.

**Figure 2:**
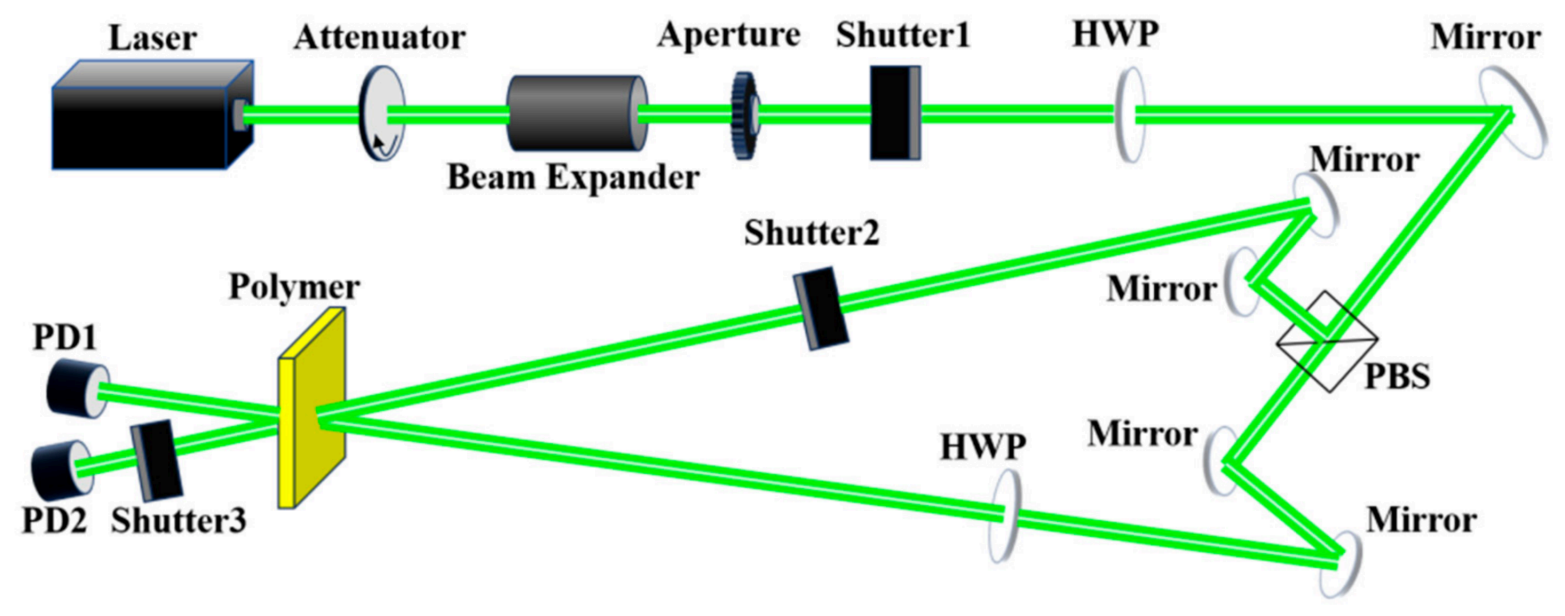
The optical path for measuring the diffraction efficiency of the material (HWP: half-wave plate; PBS: polarizing beam splitter; PD: photodetector).

Diffraction efficiency is an important indicator of holographic performance, directly affecting the clarity of information read from holographic gratings. The diffraction efficiency curve reflects the speed and strength of grating formation. Therefore, by testing the diffraction efficiency at different points on the same piece of material, the overall performance uniformity of the material can be evaluated. Diffraction efficiency *η* is calculated by the following formula [44]:

(1) $$\eta \;=\;\frac{I_{1}}{I_{0}\;+\;I_{1}}\;$$

where *I*_1_ is the intensity of the first-order diffracted light and *I*_0_ is the intensity of the transmitted light. The experimental setup is shown in Figure 2. In this experiment, the laser power was 20 mW, the beam diameter was 5 mm, and the interference angle was 24° [44]. The grating recording and reconstruction processes were controlled by shutters. During interference recording, shutter 1 and shutter 2 were opened, shutter 3 was closed, and this state was maintained for 6 s to record the interference pattern onto the material. During diffraction readout, shutter 1 was opened, shutter 2 was closed, shutter 3 was opened, and this configuration was maintained for 0.6 s to read the diffracted light from the material. This recording-and-readout cycle was repeated until the diffracted light intensity stabilized and no longer increased.

Photosensitivity (*S*) reflects the speed at which the medium records information and is an important factor affecting the recording performance of the medium. To meet the requirements of fast holographic storage, the storage medium must have high photosensitivity. The formula for calculating photosensitivity is as follows [45]:

(2) $$S\;=\;\frac{1}{\mathrm{Id}}\;(\frac{\partial \sqrt{\eta }}{\partial t})$$

where I is the recording wave intensity (0.102 W/cm^2^), d is the material thickness, and *η* is the diffraction efficiency.

According to Kogelnik’s coupled wave theory [46], the refractive index modulation (∆*n*) of the photopolymer can be calculated by

(3) $$∆n(t)=\frac{\lambda \cos\theta_{0}}{\pi d}\sin^{-1}\sqrt{\eta }\;$$

where λ is the recording wavelength (532 nm), θ_0_ is the Bragg angle between the signal beam and the reference beam, and d is the physical thickness of the material.

**Figure 3:**
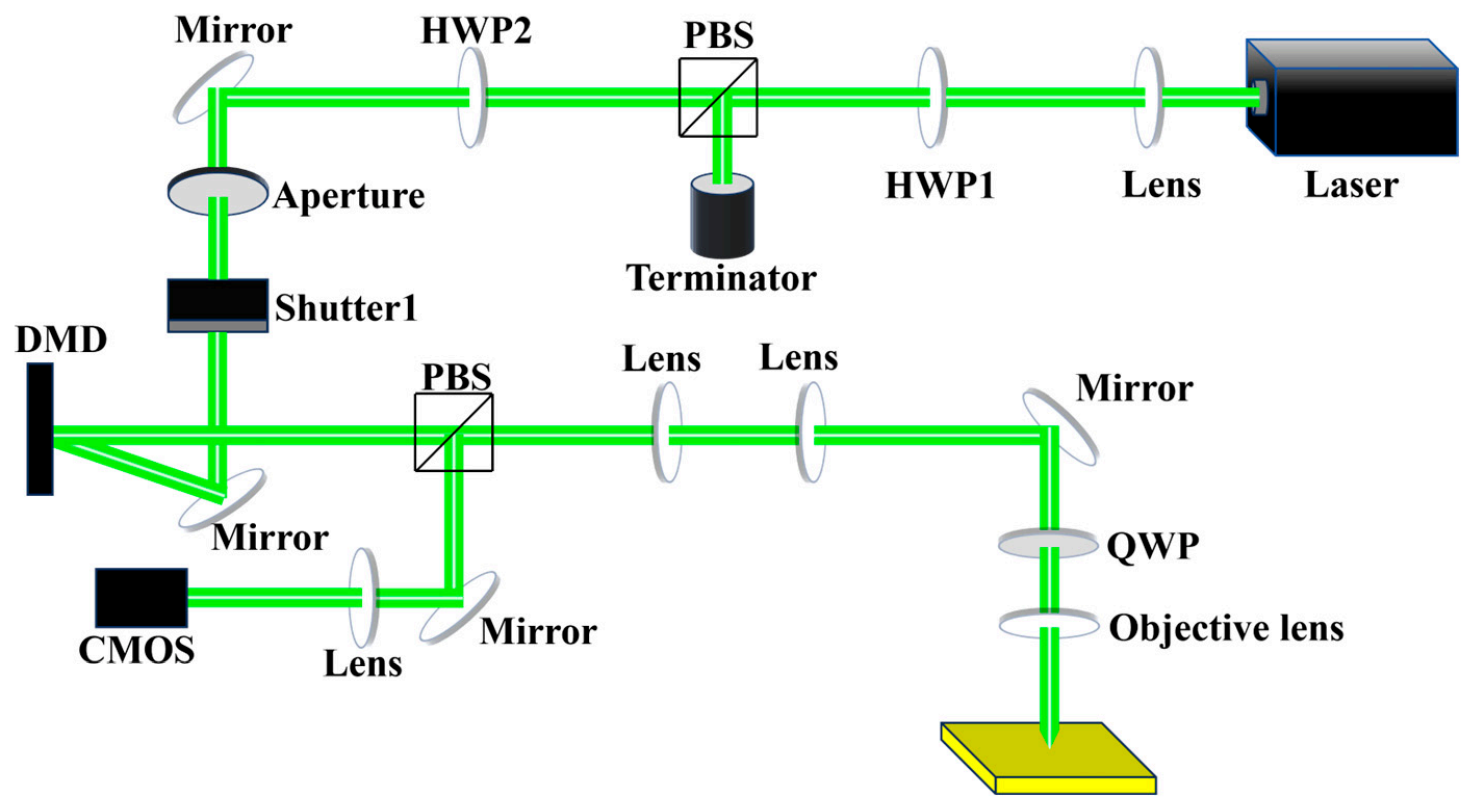
Schematic of the collinear holographic storage system (HWP: half-wave plate; QWP: quarter wave plate; PBS: polarization beam splitter; DMD: Digital Micromirror Device; CMOS: image sensor).

**Figure 4:**
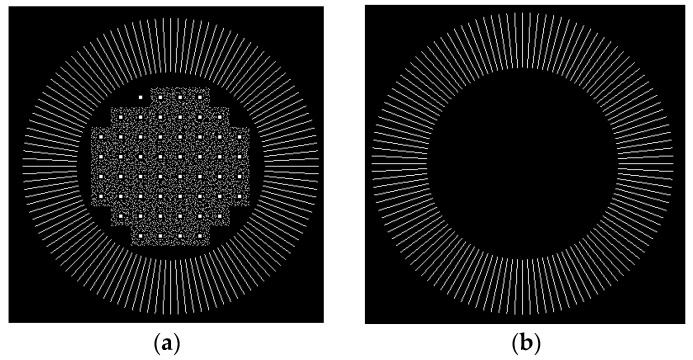
Images uploaded to DMD: (**a**) data page; (**b**) reference image.

The experimental optical path for measuring the holographic performance of the samples is shown in Figure 3. In the collinear holographic storage system illustrated in the figure, the signal beam carrying information is located at the center, while the reference beam is distributed on the peripheral circumference around the signal beam. During holographic recording, the reference pattern is loaded onto the spatial light modulator (SLM) in a manner surrounding the data page. The SLM used is an amplitude-type digital micromirror device (DMD). As shown in Figure 3, the pattern from Figure 4a is uploaded onto the spatial light modulator, where the light carrying the outer-ring pattern serves as the reference beam and the light carrying the inner information pattern serves as the signal beam. The parallel light is split into signal and reference beams by the spatial light modulator. The two beams then pass through optical components including a beam splitter, a dichroic prism, a quarter-wave plate, and an objective lens, and finally form an interference pattern in the reflective PQ/PMMA material mounted on a translation stage. During readout, the reference pattern shown in Figure 4 is loaded onto the SLM, and the reconstructed information is reflected back to the SLM through the material. The bit error rate (*BER*) and signal-to-noise ratio (*SNR*) of the reconstructed information are used to judge whether the material is suitable for holographic storage. *BER* and *SNR* are calculated as follows [47]:

(4) $$\mathrm{BER}\;=\;\frac{N_{\mathrm{error}}}{N_{\mathrm{total}}}$$

(5) $$\mathrm{SNR}\;=\frac{\mu_{\mathrm{ON}}-\mu_{\mathrm{OFF}}}{({\sigma_{\mathrm{ON}}^{2}+\sigma_{\mathrm{OFF}}^{2})}^{\frac{1}{2}}}$$

In Equation (4), N_total_ is the total number of data symbols in the original information light pattern, and *N_error_* is the number of erroneous data symbols in the reconstructed image. In Equation (5), *μ_ON_* and *μ_OFF_* represent the mean values of ON and OFF pixels, respectively, and *σ_ON_* and *σ_OFF_* represent the variances of ON and OFF pixels, respectively.

The formula for calculating the variance in this paper is given as follows:

(6) $$\mathrm{Variance}\;=\;\frac{\sum {\left(x_{i}\;-\bar{x}\;\right)}^{2}}{N}$$

where *x_i_* represents each measured value, $\bar{x}$ is the mean, and N is the total number of measurements.

## 3. Results and Discussion

### 3.1. Effect of Concentration on the Performance Uniformity of the Material

In this study, samples with a thickness of 1.0 mm were prepared, and nine different TPGDA concentrations were investigated: 0.1 wt%, 1 wt%, 10 wt%, 20 wt%, 30 wt%, 40 wt%, 50 wt%, 60 wt%, and 70 wt% (samples 2–9 in Table 2), where the total amounts of MMA and TPGDA were both 20 g. It was found that when the TPGDA concentration exceeded 50 wt%, the samples tended to detach and crack after baking (Figure S1), and 70 wt% TPGDA doping made the material too flexible, leading to easy fracture during demolding, which is unfavorable for practical holographic storage applications. Therefore, the TPGDA concentration in this study was kept below 60 wt%.

**Figure 5:**
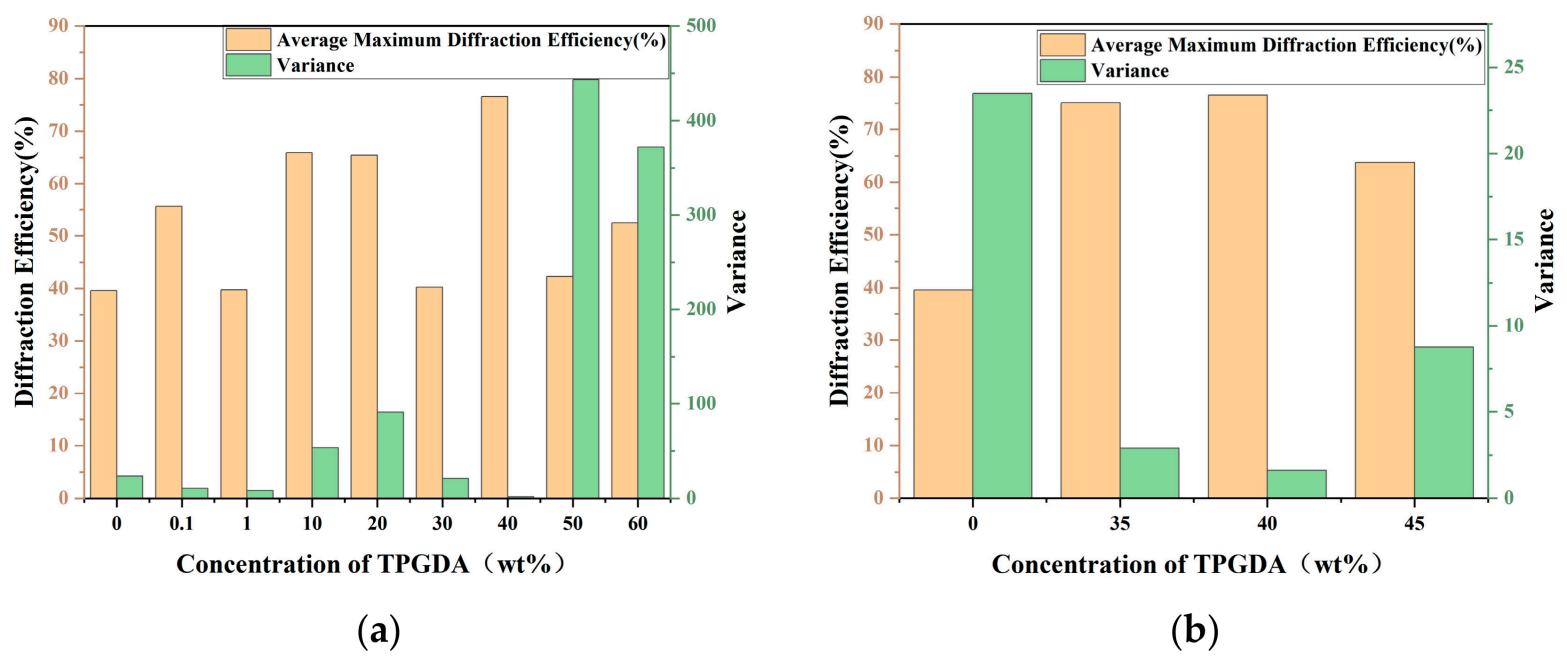
(**a**) Average diffraction efficiency and variance for TPGDA concentrations ranging from 0 to 60 wt%; (**b**) Average diffraction efficiency and variance near a TPGDA concentration of 40 wt%.

**Figure 6:**
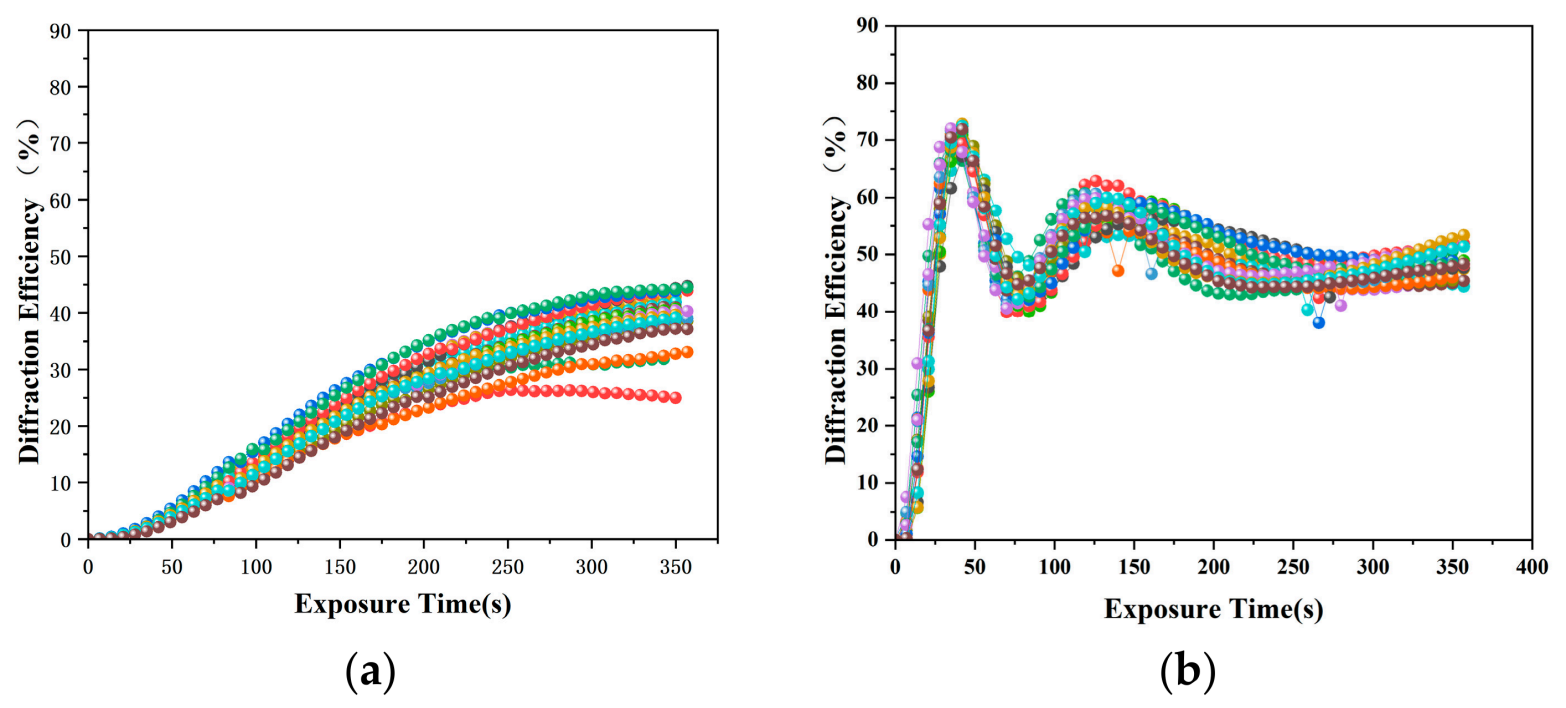
Comparison of diffraction efficiency curves between PQ/PMMA and 40 wt% TPGDA-PQ/PMMA: (**a**) diffraction efficiency curve of PQ/PMMA; (**b**) diffraction efficiency curve of PQ/PMMA doped with 40 wt% TPGDA.

For the holographic storage material PQ/PMMA, the refractive index modulation reflects the storage capacity and density. From Equation (3), the diffraction efficiency influences the refractive index modulation, and during the holographic recording process, the holographic recording capability is only related to the first attainment of the maximum diffraction efficiency. Therefore, this experiment evaluated the performance uniformity of the material by testing the maximum diffraction efficiency values at different positions on the same sample. The diffraction efficiency at each concentration was measured using the setup shown in Figure 2 and calculated using Equation (1). For each concentration, 20 points were tested, and after excluding points with bubbles or other defects, at least 17 valid data points were required for comparison. The diffraction efficiency curves for different TPGDA concentrations (Figure S2) were measured, and the average diffraction efficiency and variance at the point of maximum diffraction efficiency for each concentration were calculated, resulting in Figure 5. Detailed data are shown in Table 2. As shown in Figure 5a, over a wide concentration range (0–60 wt%), the diffraction efficiency fluctuates, and when the TPGDA concentration exceeds 40 wt%, the variance of the diffraction efficiency increases sharply, indicating that high TPGDA concentrations cause a significant deterioration in system stability. To precisely determine the optimal processing window, Figure 5b further magnifies the concentration range of 35–45 wt% (samples 10 and 11 in Table 2). Table 3 lists the specific values of the maximum average diffraction efficiency and variance for different doping ratios. The results clearly indicate that at a TPGDA concentration of 40 wt%, the material not only achieves the highest diffraction efficiency (76.58174%) but also exhibits a remarkably low diffraction efficiency variance of only 1.63574. In comparison, at a doping concentration of 35 wt%, the diffraction efficiency is similar but the variance is slightly higher; at 45 wt%, the diffraction efficiency begins to decrease and the variance increases. The diffraction efficiency tests and variance calculations show that the performance uniformity of TPGDA-PQ/PMMA doped with 40 wt% TPGDA is improved. Figure 6 compares the diffraction efficiency curves of PQ/PMMA and 40 wt% TPGDA-PQ/PMMA. (It should be noted that the different diffraction efficiency curves within a single figure originate from distinct areas of the same sample material, and this convention is followed for all subsequent diffraction efficiency figures presented herein.) Compared with PQ/PMMA, the maximum diffraction efficiency is increased by 1.95-fold, and the time to reach the maximum diffraction efficiency is reduced to 1/6 of that of PQ/PMMA.

**Figure 7:**
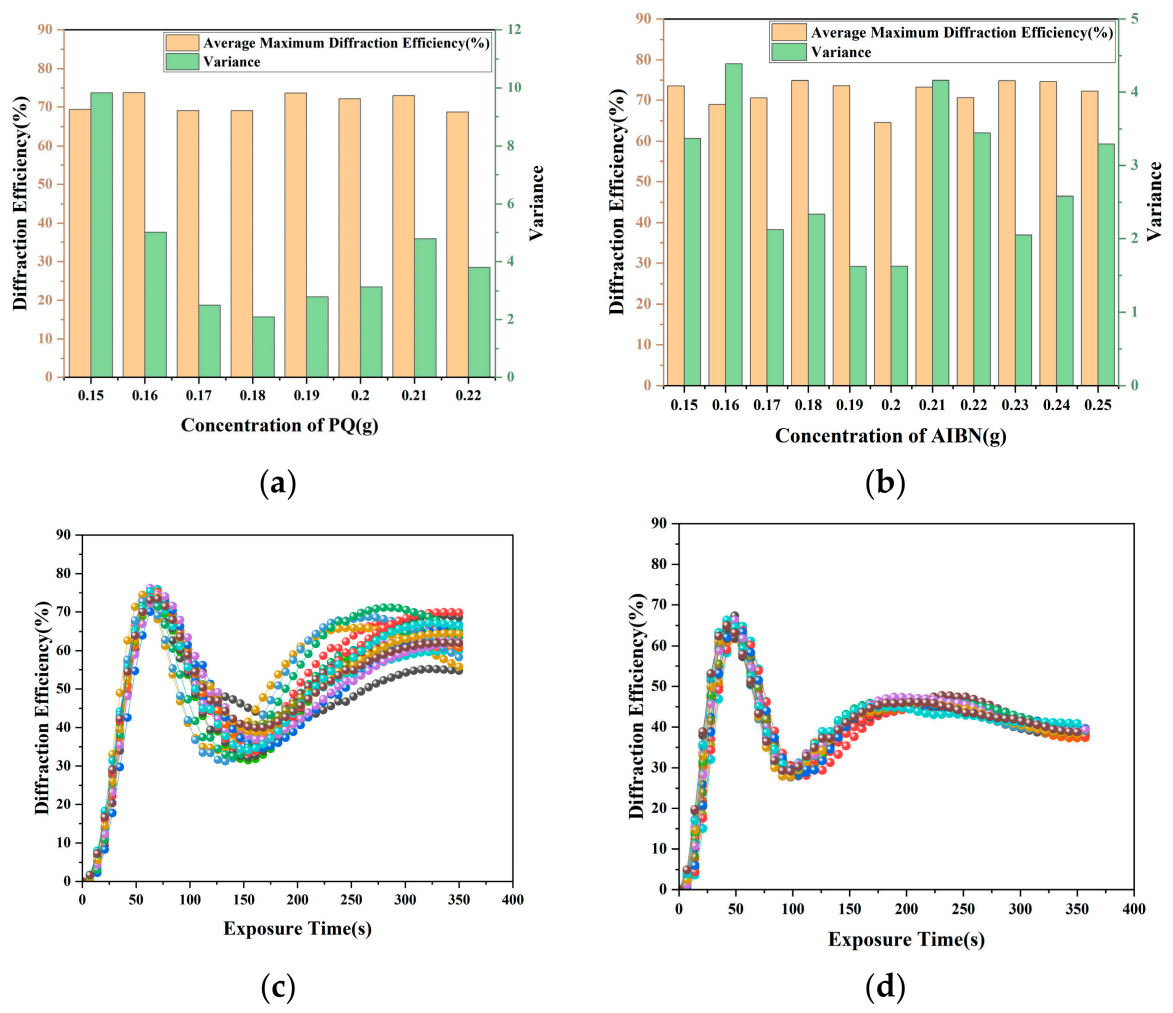
(**a**) Average diffraction efficiency and variance of samples with different PQ contents; (**b**) average diffraction efficiency and variance of samples with different AIBN contents; (**c**) diffraction efficiency curve of the sample with 0.19 g AIBN; (**d**) diffraction efficiency curve of the sample with 0.20 g AIBN.

Based on the introduction of 40 wt% TPGDA, the optimal preparation ratio was further investigated by varying the amounts of PQ and AIBN. First, to explore the effect of PQ content on the performance uniformity of the PQ/PMMA holographic storage material, a series of samples with different PQ contents were prepared while keeping the AIBN amount constant. The PQ content was increased from 0.15 g to 0.25 g in increments of 0.01 g. It was observed that when the PQ content reached 0.23 g, precipitation began to occur; therefore, only samples with a PQ content not exceeding 0.22 g were tested (Figure S3). The diffraction efficiency of each sample during holographic recording was recorded using the setup in Figure 2, and the average diffraction efficiency and variance at the maximum diffraction efficiency were calculated to evaluate material uniformity. The results are shown in Figure 7a. It can be seen that the average diffraction efficiencies of samples with different PQ contents are similar. Among them, the sample with a PQ content of 0.18 g exhibits the smallest variance, indicating the best performance uniformity. Therefore, 0.18 g was determined as the optimal PQ amount. Next, with the PQ content fixed at 0.18 g, the AIBN content was increased from 0.15 g to 0.25 g in increments of 0.01 g. The samples were again tested using the diffraction efficiency setup (Figure S4). The average diffraction efficiency and variance at the maximum diffraction efficiency for each sample were calculated, and the results are presented in Figure 7b. From Figure 7b, it can be seen that the variances of the samples with AIBN contents of 0.19 g and 0.20 g are both small and close in value, but the average diffraction efficiency of the sample with 0.20 g AIBN is slightly lower than that with 0.19 g AIBN. Further examination of the overall diffraction efficiency curves (Figure 7c,d) reveals that the sample with 0.20 g AIBN exhibits a more uniform trend of diffraction efficiency change throughout the recording process. Considering both the maximum diffraction efficiency and uniformity, and to maintain a material with high maximum diffraction efficiency and good uniformity for subsequent studies, the sample with 0.20 g AIBN was selected as the preparation ratio with better performance uniformity. Through the above studies, it was confirmed that the introduction of TPGDA can improve the performance uniformity of PQ/PMMA. The formulation for preparing uniform TPGDA-PQ/PMMA is TPGDA:MMA:AIBN:PQ = 8 g:12 g:0.20 g:0.18 g.

### 3.2. Effect of Post-Curing Process on the Diffraction Efficiency and Stability of TPGDA-PQ/PMMA

**Figure 8:**
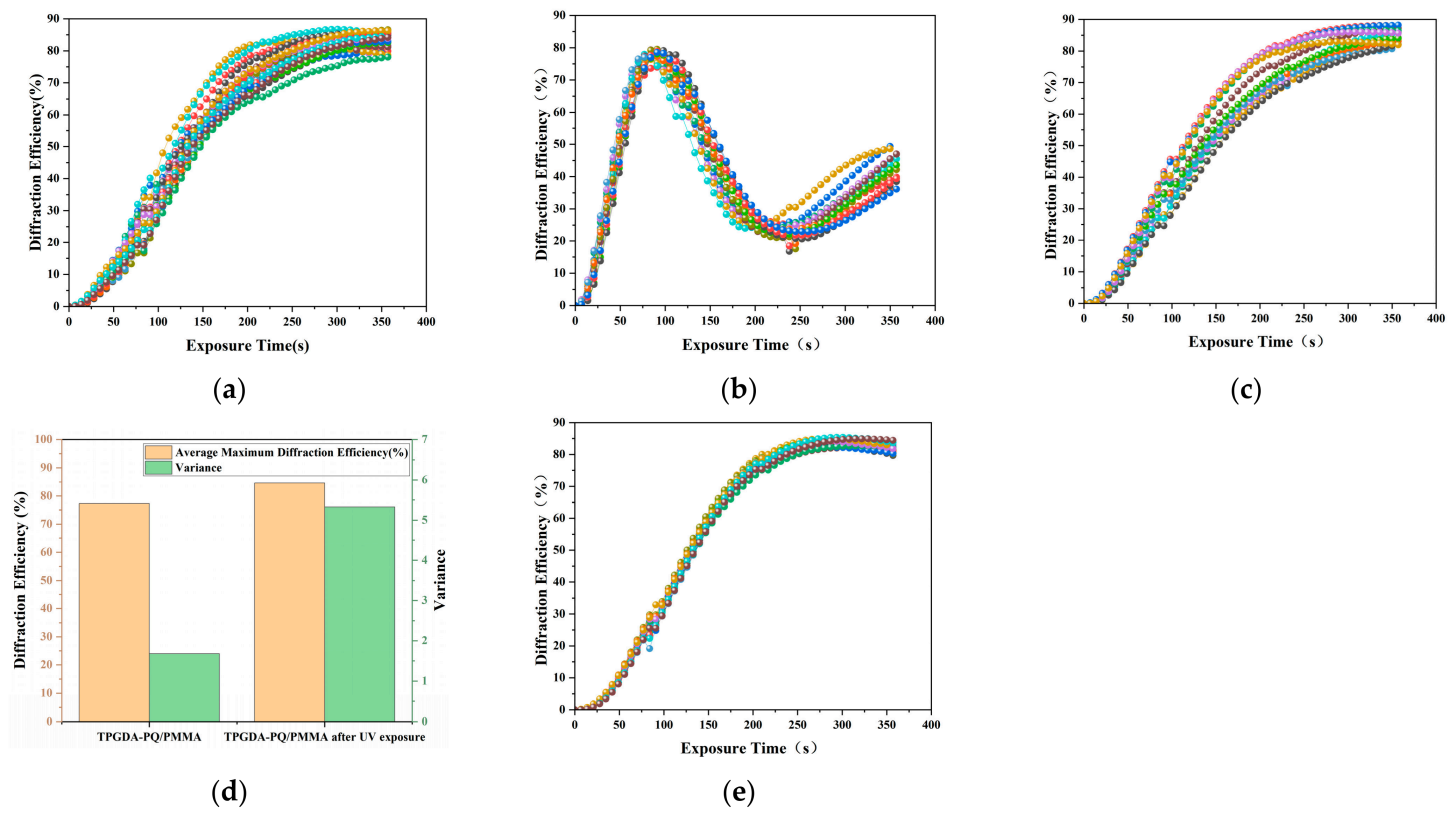
Diffraction efficiency test results of the optimally formulated sample: (**a**) TPGDA-PQ/PMMA prepared by baking at 70 °C for 20 h; (**b**) untreated 1.0 mm TPGDA-PQ/PMMA; (**c**) 1.0 mm TPGDA-PQ/PMMA after 100 s UV irradiation; (**d**) average diffraction efficiency and variance before and after UV curing of TPGDA-PQ/PMMA; (**e**) untreated 0.5 mm TPGDA-PQ/PMMA.

It was observed that the diffraction efficiency of samples after TPGDA introduction always showed a trend of first increasing, then decreasing, and then increasing again to a plateau. This was first attributed to incomplete curing of the material during thermal polymerization after TPGDA introduction. In the process of diffraction efficiency testing, photocuring of the material may occur under laser illumination, which could result in fluctuations of the diffraction efficiency. This study first attempted to achieve complete curing of the samples by thermal polymerization. Extending the baking time of 1.0 mm samples at 60 °C from 20 h to 48 h still did not allow the samples to maintain the maximum diffraction efficiency, indicating that the curing degree of thick TPGDA-doped samples under thermal polymerization conditions is limited. With a thermal polymerization time of 20 h, the polymerization temperature was increased. The experimental results showed that the sample prepared by thermal polymerization at 70 °C for 20 h exhibited the highest diffraction efficiency (see Figure 8a) and good uniformity (Figure S5). However, this sample developed cracks after being placed at room temperature for one day (Figure S6). Further increasing the polymerization temperature (up to 85 °C) or extending the polymerization time (up to 25 h) not only accelerated the cracking process but also generated a large number of bubbles in the prepared sample, rendering it unusable. These results indicate that while increasing the temperature accelerates curing, the process is too violent and easily induces thermal stress cracking. On the other hand, simply extending the thermal polymerization time at a lower temperature fails to completely consume the residual double bonds in the system because the free radical generation rate of the thermal initiator AIBN is limited, resulting in incomplete curing. Studies have shown that in photopolymer systems, photopolymerization and thermal polymerization follow distinctly different initiation mechanisms. Thermal polymerization relies on the decomposition of AIBN at high temperatures to generate primary free radicals, which then initiate the chain polymerization of MMA monomers to form the PMMA matrix. In contrast, UV curing uses ultraviolet light to excite the photosensitizer PQ in the system, generating active free radicals that initiate the crosslinking polymerization of the remaining acrylate double bonds. Based on the above mechanistic differences and the fact that UV curing can be carried out at room temperature with a relatively mild reaction, this study introduced a UV curing treatment. This treatment aims to promote deep crosslinking of residual double bonds without inducing excessive thermal stress, thereby stabilizing the internal structure of the material. Half of the prepared TPGDA-PQ/PMMA sample was shielded and subjected to UV curing. The UV light has a wavelength of 395 nm, and the UV lamp power incident on the sample surface is in the range of 12–13.5 mW. By comparing the diffraction efficiency test results (Figure 8b,c), it was found that the sample after 100 s of UV curing could maintain the maximum diffraction efficiency. The average diffraction efficiency and variance at the maximum diffraction efficiency before and after UV curing were calculated and are shown in Figure 8d. As seen from Figure 8d, the diffraction efficiency increased after UV curing, but the variance increased from 1.68 before UV curing to 5.32 after UV curing, indicating a slight reduction in uniformity. Nevertheless, compared with the variance of the maximum diffraction efficiency of PQ/PMMA (23.49), the UV-cured sample still exhibited good uniformity. Previous studies have shown that for the PQ/PMMA system, a smaller sample thickness allows correspondingly lower thermal curing temperature or shorter curing time. In other words, under the same curing temperature and time, thinner samples are easier to fully cure. Based on this, in this study, TPGDA-PQ/PMMA samples with a thickness of 0.5 mm were prepared under the condition of thermal polymerization at 60 °C for 20 h. The diffraction efficiency test (Figure 8e) once again indicates that the decrease in diffraction efficiency observed in the 1.0 mm samples is due to incomplete curing. Therefore, to maintain stable diffraction efficiency, effective methods include UV curing of the samples and reducing the thickness.

**Figure 9:**
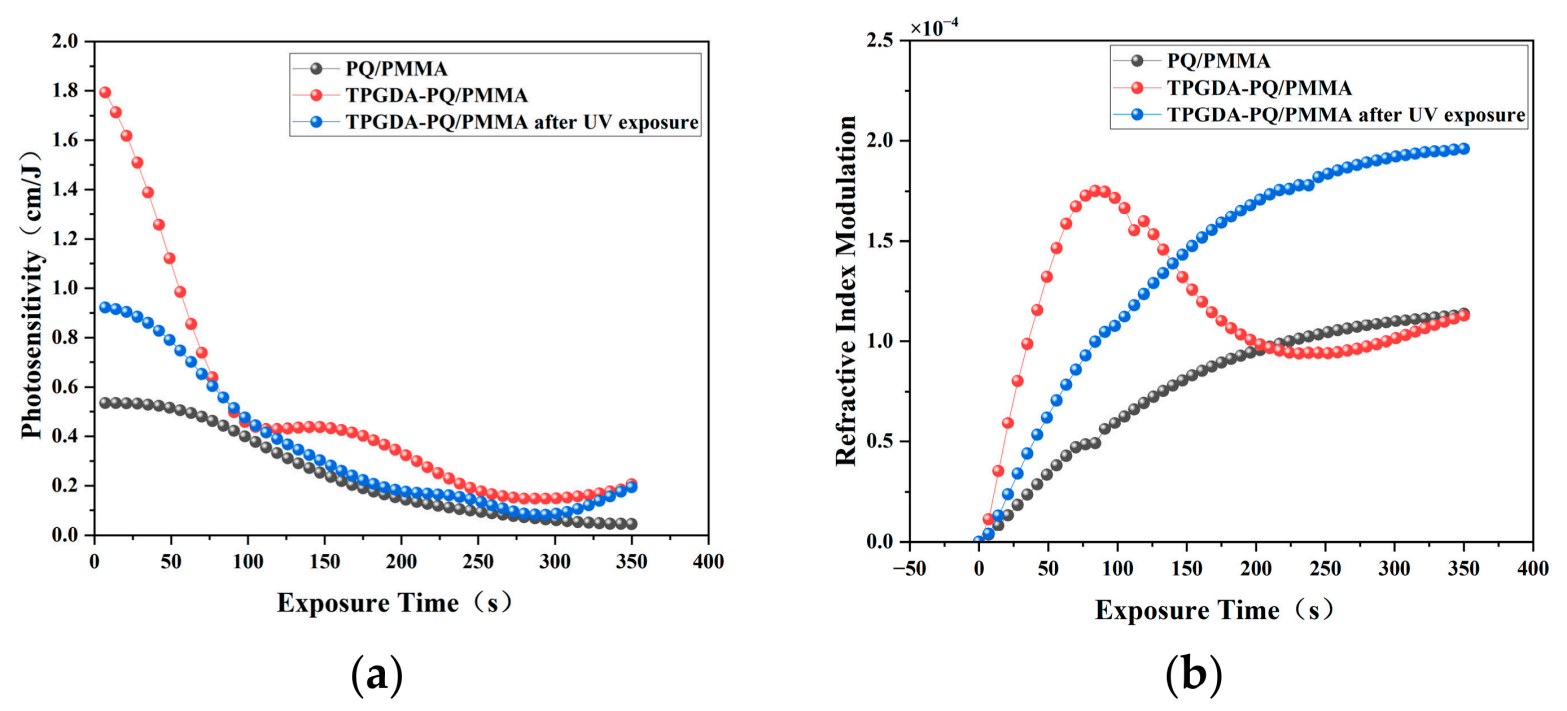
(**a**) Photosensitivity of samples with different treatment methods; (**b**) refractive index modulation of samples with different treatment methods.

Using the optical setup shown in Figure 2, the photosensitivity and refractive index modulation of the 1.0 mm samples prepared under different treatment conditions were calculated using Equations (2) and (3) (Figure 9). The TPGDA-PQ/PMMA sample before 100 s UV exposure exhibited the highest photosensitivity. After UV curing, the photosensitivity of TPGDA-PQ/PMMA was lower than that before UV curing but still higher than that of the pristine PQ/PMMA. This is consistent with the rate of diffraction efficiency growth. The refractive index modulation of TPGDA-PQ/PMMA reached a maximum of 1.75 × 10^−4^ cm/J, which is approximately 1.6 times higher than that of PQ/PMMA (1.12 × 10^−4^ cm/J). After reaching the maximum, it decreased to around 1.12 × 10^−4^ cm/J. The refractive index modulation of TPGDA-PQ/PMMA after 100 s UV curing reached 1.96 × 10^−4^ cm/J, which is 1.75 times higher than that of PQ/PMMA. This breakthrough is of great significance for holographic recording in the PQ/PMMA polymer system.

### 3.3. Microscopic Mechanism Characterization of TPGDA-PQ/PMMA

**Figure 10:**
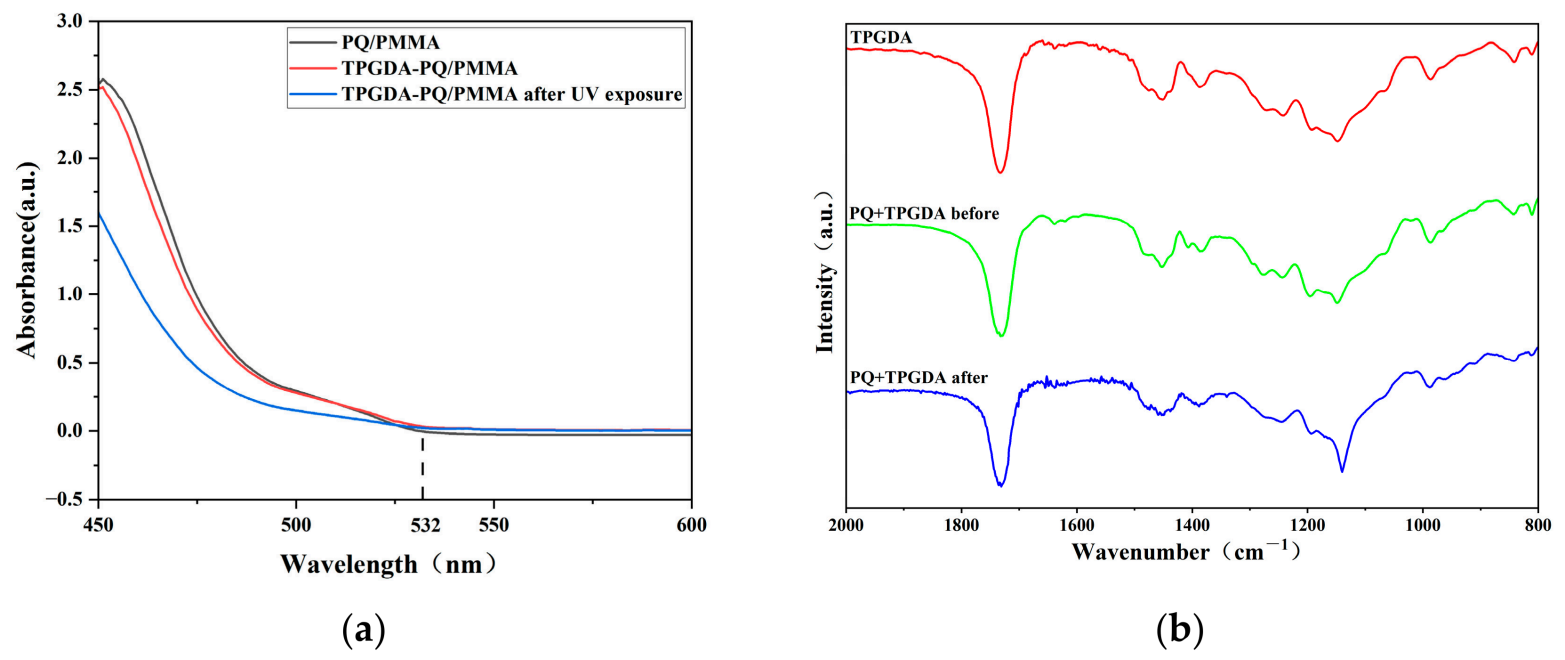
(**a**) Visible light absorption spectra; (**b**) FT-IR spectra.

To elucidate the positive effect of TPGDA on the performance uniformity of PQ/PMMA from a microscopic perspective, characterization experiments were performed on the TPGDA-PQ/PMMA material. The results are shown in Figure 10.

The visible light absorption spectra of the TPGDA-PQ/PMMA material are shown in Figure 10a. The absorption coefficient curves of the TPGDA-doped samples remain generally consistent, indicating that the introduction of TPGDA does not significantly affect the light absorption of the raw materials. The samples exhibit minimal absorption only at the wavelength of 532 nm. This result confirms that the use of a 532 nm green laser for holographic recording in this experiment is reasonable, as this wavelength lies in the lowest absorption region of the material, effectively avoiding energy loss and adverse effects on the recording process due to excessive light absorption.

FT-IR measurements were performed on the TPGDA-PQ/PMMA material, and the results are shown in Figure 10b. Comparing the curves of PQ/PMMA and TPGDA-PQ/PMMA, no new peaks appear, suggesting that the introduction of TPGDA does not induce reactions with the PMMA chains. Comparing the curves of TPGDA-PQ/PMMA and UV-irradiated TPGDA-PQ/PMMA, the positions, shapes, and relative intensities of the absorption peaks are essentially identical, and no new characteristic absorption peaks appear, indicating that the short-time UV post-curing treatment does not initiate new chemical reactions or functional group transformations. This result further confirms that the primary role of UV curing is to promote further crosslinking of residual double bonds in the system, rather than altering the chemical bonding state between TPGDA and the PMMA matrix. The performance of PQ/PMMA is mainly determined during the photoreaction stage. Under illumination, PQ absorbs photon energy and is excited to the singlet excited state ^1^PQ. The unstable ^1^PQ gradually transforms into the more stable triplet excited state ^3^PQ. The C=O bond on ^3^PQ breaks and reacts with the C=C bond on PMMA to generate a semiquinone radical and a free radical, followed by further photolinkage reactions to produce PQ/PMMA, ultimately exhibiting different diffraction efficiency performances [18,48,49]. In the FT-IR spectra, the stretching vibration of the C=O bond corresponds to 1725 cm^−1^, and that of the C=C bond corresponds to 1640 cm^−1^. A single sample was cut into nine different regions (Figure S7), and FT-IR measurements were performed on each region. The ratio of C=C to C=O bonds in the nine regions was calculated, and the variance of these ratios was computed. By comparison (Table 4), the TPGDA-PQ/PMMA shows the best uniformity at the microscopic level, followed by the UV-irradiated TPGDA-PQ/PMMA, and finally the pristine PQ/PMMA, which is consistent with the measured diffraction efficiency uniformity.

### 3.4. Collinear Holographic Information Recording of TPGDA-PQ/PMMA

The collinear holographic data storage system is characterized by compactness and strong compatibility. Its working principle involves splitting a beam of light into two parts: an information beam and a reference beam. As shown in Figure 4, the outer ring represents the reference beam, and the central part is the data page carrying the modulated information. The data page consists of a synchronization mark in the upper left corner and 51 data sub-pages. Each sub-page contains 32 data symbols and a sub-page synchronization mark. All symbols within the data page are extracted sequentially and compared with the original image. Matched symbols are considered correct, while mismatched ones are considered incorrect. During the information storage stage, the two beams are used for interference recording, and diffraction readout is performed with the reference beam. The optical path is shown in Figure 3. During recording, the data are first encoded, and the encoded data page is loaded onto the optical path using a digital micromirror device (DMD). Finally, interference recording occurs near the focal plane of the objective lens. During the information readout stage, only the reference beam on the outer periphery needs to be loaded onto the DMD to reconstruct the image of the signal light. The *BER* and *SNR* of the reconstructed image are calculated using Equations (4) and (5).

**Figure 11:**
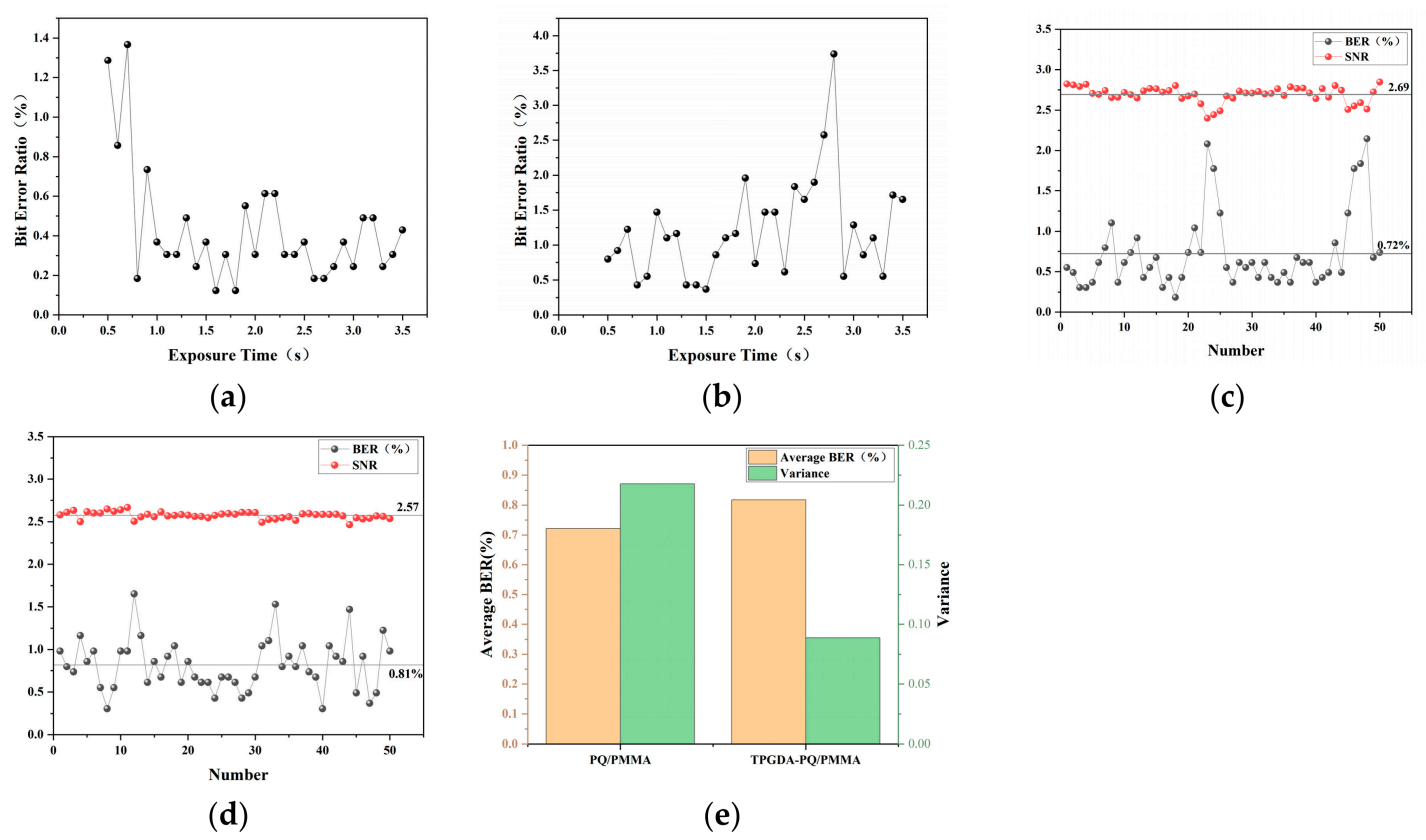
(**a**) *BER* of PQ/PMMA recorded information at different recording times; (**b**) *BER* of TPGDA-PQ/PMMA recorded information at different recording times; (**c**) *BER* and *SNR* of PQ/PMMA recording 50 pieces of information at a recording time of 1.8 s; (**d**) *BER* and *SNR* of TPGDA-PQ/PMMA recording 50 pieces of information at a recording time of 0.8 s; (**e**) average *BER* and variance for 50 recording points of PQ/PMMA and TPGDA-PQ/PMMA.

We prepared yellow sheet materials (50 mm × 50 mm × 0.5 mm) of PQ/PMMA and 40 wt% TPGDA-PQ/PMMA with a reflective mirror. Previous experiments showed that the diffraction efficiency of TPGDA-PQ/PMMA does not decrease at a thickness of 0.5 mm (Figure 8e); therefore, stable diffraction efficiency can be obtained without additional UV curing, and the materials can be used after thermal curing. These materials were tested on the collinear holographic data storage system. The recording time was varied from 0.5 s to 3.5 s in increments of 0.1 s. The test results obtained from the collinear holographic data storage system are shown in Figure 11a,b. From these figures, it can be seen that the optimal recording time for PQ/PMMA is 1.8 s, while that for TPGDA-PQ/PMMA is 0.8 s. Figure 11c,d present the experimental results of recording 50 points with PQ/PMMA and TPGDA-PQ/PMMA at different recording times and at the optimal recording time. The average *BER* and variance of the two samples were calculated and are shown in Figure 11e. The average *BER* of TPGDA-PQ/PMMA is 0.81%, which is only 0.09% higher than that of PQ/PMMA. The average *SNR* is 2.57, which is 0.12 lower than that of PQ/PMMA. The *BER* variance of TPGDA-PQ/PMMA is 0.08867, which is approximately 40% lower than that of PQ/PMMA (0.21763), once again verifying that the introduction of TPGDA into PQ/PMMA improves the uniformity of holographic recording. Therefore, the TPGDA-doped PQ/PMMA photopolymer not only possesses the capability for holographic information recording and readout but also exhibits an increased recording/readout speed.

## 4. Conclusions

This study addressed the problem of insufficient performance uniformity of PQ/PMMA holographic storage materials in practical applications by introducing a low-viscosity reactive diluent, TPGDA, and systematically investigated its influence on the consistency of the microstructure and macroscopic holographic performance. By optimizing the TPGDA doping concentration and the amounts of PQ and AIBN, the optimal formulation was determined to be TPGDA:MMA:AIBN:PQ = 8 g:12 g:0.20 g:0.18 g, with an optimal TPGDA doping concentration of 40 wt%. Under these conditions, the maximum diffraction efficiency of the material reached 76.58%, and the variance of diffraction efficiency over 20 different test points was only 1.64, far lower than the value of 23.49 for the undoped sample, indicating that TPGDA significantly improves the performance uniformity of the material during holographic recording. FT-IR characterization results showed that after TPGDA doping, the variance of the absorbance ratio of C=C to C=O bonds in different regions of the material decreased significantly, and the uniformity of microscopic functional group distribution was improved, providing a molecular-level explanation for the enhancement of performance uniformity. Collinear holographic storage experiments further demonstrated that the optimal recording time of TPGDA-PQ/PMMA is shortened from 1.8 s (undoped) to 0.8 s, corresponding to a recording speed increase of approximately 2-fold. The average bit error rate is 0.81%, and the *BER* variance is 0.08867, which is reduced by about 40% compared with that of the undoped sample (0.21763), confirming that the readout consistency and reliability of the material in holographic data storage are effectively enhanced. In summary, reducing the system viscosity by introducing TPGDA to promote molecular diffusion and uniform distribution of functional groups is an effective strategy for improving the performance uniformity of PQ/PMMA holographic storage materials. This study provides experimental evidence and theoretical guidance for the development of highly uniform and reliable photopolymer holographic recording media.

## Figures and Tables

**Table 1:** Details of reagents used in the experiment.

Name	Molecular Formula	Molecular Weight	State	Purity	Manufacturer
Methyl methacrylate (MMA)	C_5_H_8_O_2_	100.12	Liquid	>99.5%	Macklin, Shanghai, China
2,2-Azobisisobutyronitrile (AIBN)	C_8_H_12_N_4_	164.21	solid	99%	
Dipropylene glycol diacrylate (TPGDA)	C_15_H_24_O_6_	300.35	Liquid	90%	
Phenanthrenequinone (PQ)	C_14_H_8_O_2_	208.21	solid	>98.1%	TCI, Shanghai, China

**Table 2:** Content of each component in the prepared samples.

Number	Concentration of TPGDA (wt%)	TPGDA (g)	MMA (g)	AIBN (g)	PQ (g)
1	0	0.00	20.00	0.20	0.20
2	0.1	0.02	19.98	0.20	0.20
3	1	0.20	19.80	0.20	0.20
4	10	2.00	18.00	0.20	0.20
5	20	4.00	16.00	0.20	0.20
6	30	6.00	14.00	0.20	0.20
7	40	8.00	12.00	0.20	0.20
8	50	10.00	10.00	0.20	0.20
9	60	12.00	8.00	0.20	0.20
10	35	7.00	13.00	0.20	0.20
11	45	9.00	11.00	0.20	0.20
12	40	8.00	12.00	0.20	0.15
13	40	8.00	12.00	0.20	0.16
14	40	8.00	12.00	0.20	0.17
15	40	8.00	12.00	0.20	0.18
16	40	8.00	12.00	0.20	0.19
17	40	8.00	12.00	0.20	0.21
18	40	8.00	12.00	0.20	0.22
19	40	8.00	12.00	0.20	0.23
20	40	8.00	12.00	0.20	0.24
21	40	8.00	12.00	0.20	0.25
22	40	8.00	12.00	0.15	0.18
23	40	8.00	12.00	0.16	0.18
24	40	8.00	12.00	0.17	0.18
25	40	8.00	12.00	0.18	0.18
26	40	8.00	12.00	0.19	0.18
27	40	8.00	12.00	0.21	0.18
28	40	8.00	12.00	0.22	0.18
29	40	8.00	12.00	0.23	0.18
30	40	8.00	12.00	0.24	0.18
31	40	8.00	12.00	0.25	0.18

**Table 3:** Average diffraction efficiency and standard deviation at different TPGDA concentrations.

Doping Concentration (wt%)	Optimal Diffraction Efficiency (%)	Variance
0	39.57959	23.48554
0.1	55.62074	10.31373
1	39.71337	8.05929
10	65.86635	53.39288
20	65.41365	91.02969
30	40.25502	20.78032
35	75.10809	2.90565
40	76.58174	1.63574
45	63.72996	8.75884
50	42.30207	443.30544
60	52.47116	371.93502

**Table 4:** Ratios of C=C to C=O from FT-IR tests.

	Transmittance of C=C (%)	Transmittance of C=O (%)	Ratio	Ratio Variance
PQ/PMMA	43.90	25.10	1.7490	0.0454
	70.60	61.00	1.1574	
	56.30	46.20	1.2186	
	61.00	40.10	1.5212	
	60.00	47.20	1.2712	
	84.50	73.20	1.1544	
	71.80	58.80	1.2211	
	80.20	69.30	1.1573	
	79.50	72.20	1.1011	
TPGDA-PQ/PMMA	73.10	54.80	1.3339	0.0061
	75.70	53.90	1.4045	
	72.70	53.60	1.3563	
	69.90	58.30	1.1990	
	61.30	52.10	1.1766	
	68.60	56.10	1.2228	
	83.70	68.60	1.2201	
	78.00	62.00	1.2581	
	84.40	65.30	1.2925	
TPGDA-PQ/PMMA after UV exposure	65.60	47.00	1.3957	0.0185
	74.20	54.20	1.3690	
	77.10	63.70	1.2104	
	73.40	62.60	1.1725	
	49.00	37.70	1.2997	
	58.10	43.80	1.3265	
	37.30	24.90	1.4980	
	49.40	31.70	1.5584	
	63.30	41.50	1.5253	

## Data Availability

The original contributions presented in this study are included in the article/Supplementary Materials. Further inquiries can be directed to the corresponding author.

## Supplementary Materials

The following supporting information can be downloaded at: https://www.mdpi.com/article/10.3390/polym18151851/s1, Figure S1: Cracking and falling off after baking at high concentration; Figure S2: Diffraction efficiency plots with different TPGDA concentrations: (a) 0 wt%; (b) 0.1 wt%; (c) 1 wt%; (d) 10 wt%; (e) 20 wt%; (f) 30 wt%; (g) 40 wt%; (h) 50 wt%; (i) 60 wt%; Figure S3: Diffraction efficiency charts measured with different PQ for 0.2 g of AIBN: (a) 0.15 g; (b) 0.16 g; (c) 0.17 g; (d) 0.18 g; (e) 0.19 g; (f) 0.20 g; (g) 0.21 g; (h) 0.22 g; Figure S4: Diffraction efficiency charts measured with different AIBN for 0.18 g of PQ: (a) 0.15 g; (b) 0.16 g; (c) 0.17 g; (d) 0.18 g; (e) 0.19 g; (f) 0.20 g; (g) 0.21 g; (h) 0.22 g; (i) 0.23 g; (j) 0.24 g; (k) 0.25 g; Figure S5: Diffraction efficiency spectra of TPGDA-PQ/PMMA prepared at different temperatures: (a) 68 °C; (b) 69 °C; (c) 70 °C; (d) 71 °C; (e) 72 °C; Figure S6: Samples prepared under high-temperature conditions gradually crack after being placed for a period of time; Figure S7: Nine regions for sample division.
