# Supplementary material for: Effect of Tripropylene Glycol Diacrylate Doping on the Uniformity of Phenanthrenequinone/Poly(Methyl Methacrylate) Photopolymer
Source: Polymers (Basel). 2026 Jul 28;18(15):1851. doi: 10.3390/polym18151851 (PMC13468308; doi:10.3390/polym18151851)
Supplement: Supplementary file 1 [file polymers-18-01851-s001.zip › polymers-4436363-supplementary.pdf]

Supporting Information

Effect of TPGDA Doping on Performance Uniformity of PQ/PMMA Holographic Storage Materials

Enqiang Wu [1], Junhui Wu [1], Shenghui Ke [1], Jianlei Li [1], Erkang Yang [1], Xuelin Wang [1], Jun Xie [1], Jianwei Wu [1] and Xiaodi Tan [2,*]

1 College of Photonic and Electronic Engineering, Fujian Normal University, Fuzhou 350117, China
2 Information Photonics Research Center, Key Laboratory of Optoelectronic Science and for Medicine of Ministry of Education, Fujian Provincial Key Laboratory of Photonics Technology, Fujian Provincial Engineering Technology Research Center of Photoelectric Sensing Application, Fujian Normal University, Fuzhou 350117, China
* Correspondence: xtan@fjnu.edu.cn

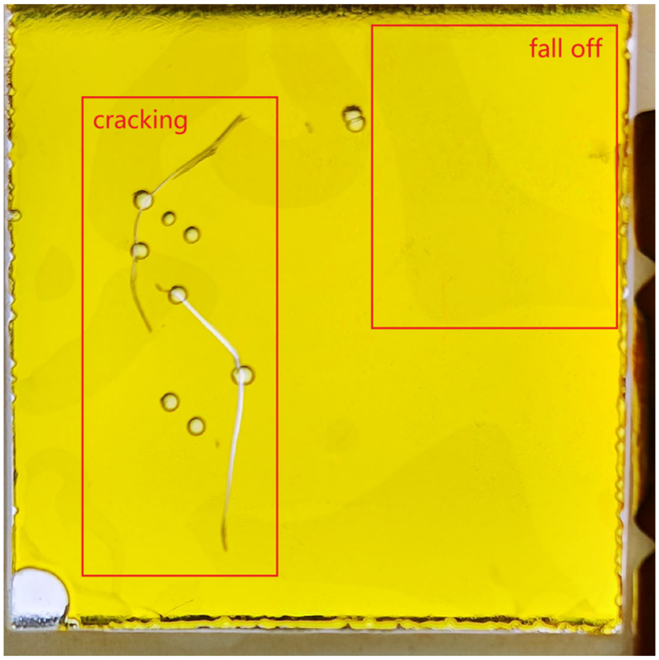

Figure S1. Cracking and falling off after baking at high concentration.

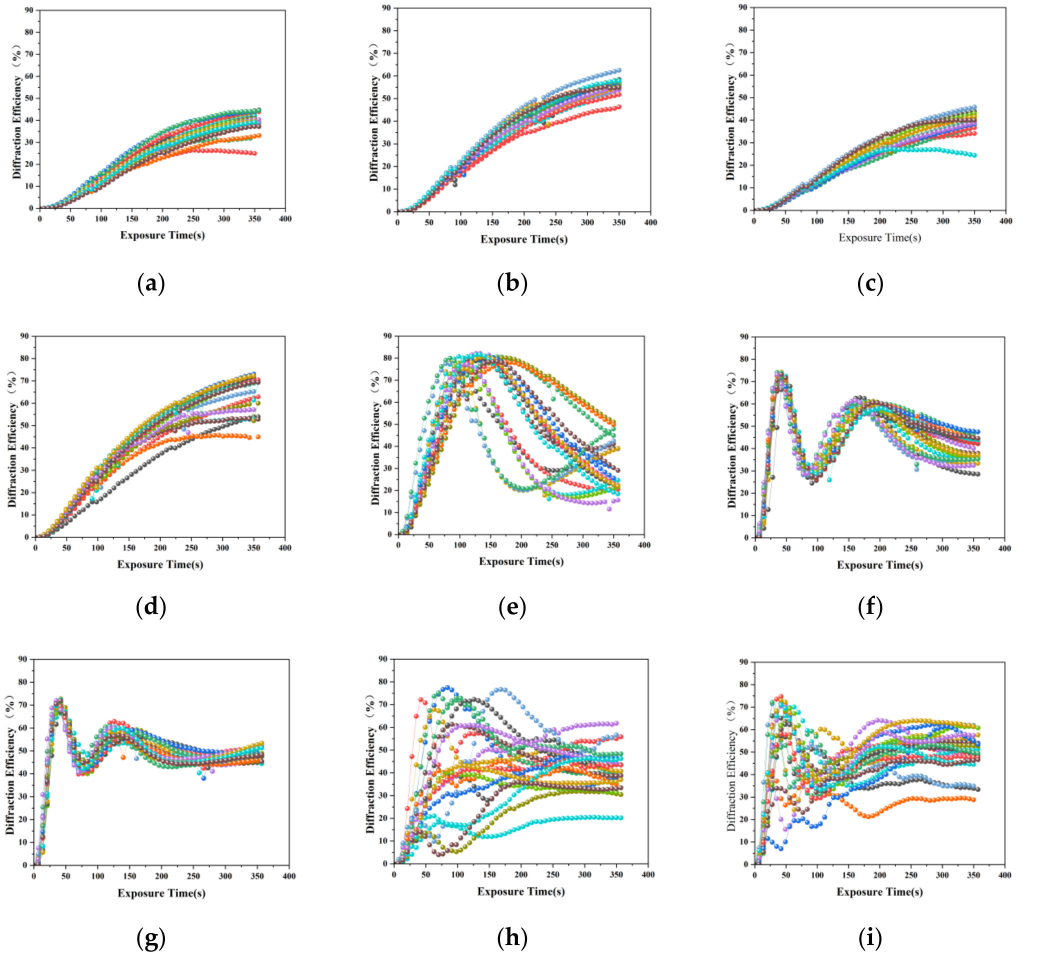

Figure S2. Diffraction efficiency plots with different TPGDA concentrations: (a) 0 wt%; (b) 0.1 wt%; (c) 1 wt%; (d) 10 wt%; (e) 20 wt%; (f) 30 wt%; (g) 40 wt%; (h) 50 wt%; (i) 60 wt%.

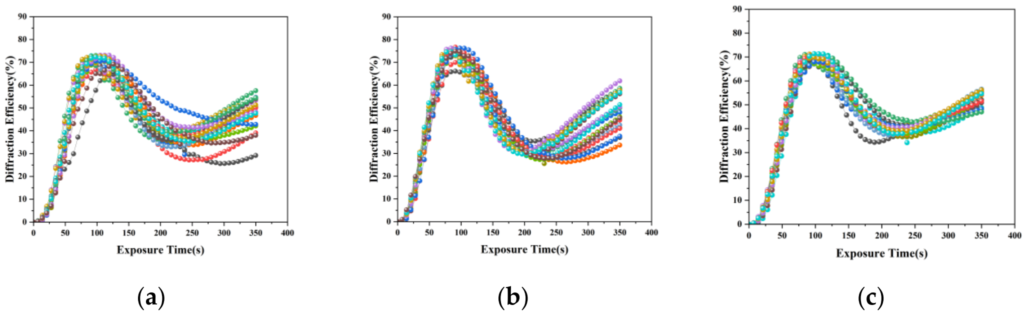

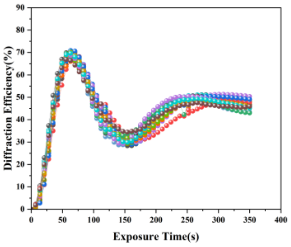

(**d**)

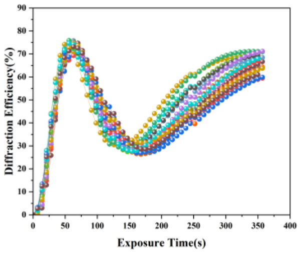

(**e**)

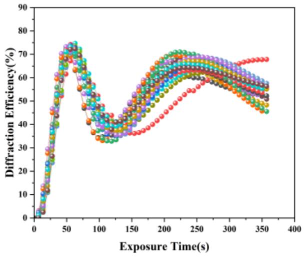

(**f**)

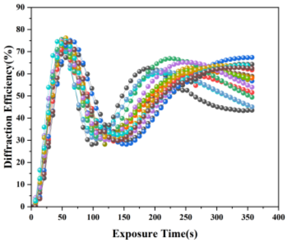

(**g**)

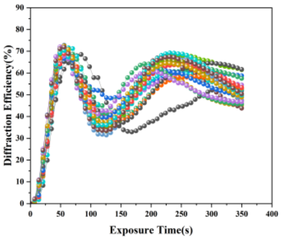

(**h**)

Figure S3. Diffraction efficiency charts measured with different PQ for 0.2 g of AIBN: (a) 0.15 g; (b) 0.16 g; (c) 0.17 g; (d) 0.18 g; (e) 0.19 g; (f) 0.20 g; (g) 0.21 g; (h) 0.22 g.

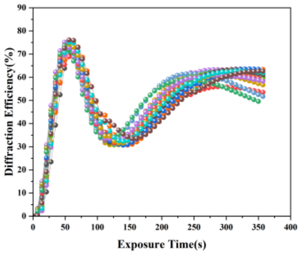

(**a**)

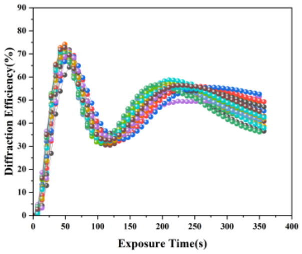

(**b**)

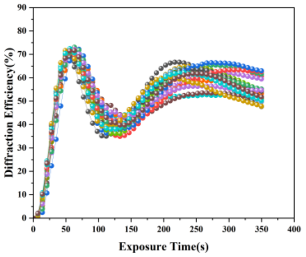

(**c**)

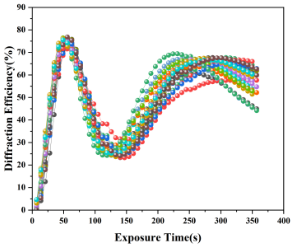

(**d**)

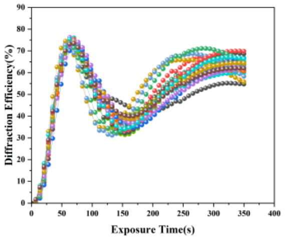

(**e**)

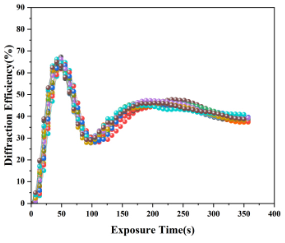

(**f**)

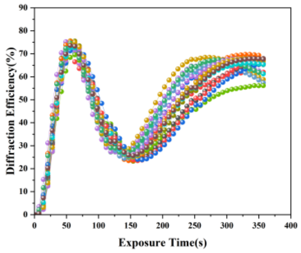

(**g**)

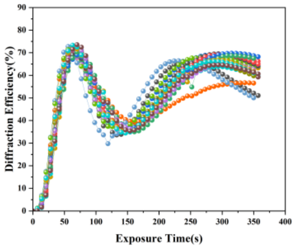

(**h**)

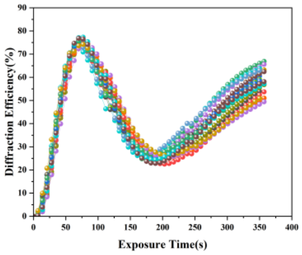

(**i**)

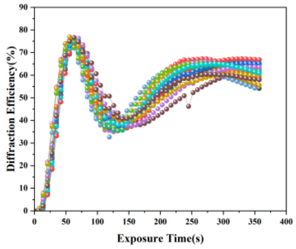

(j)

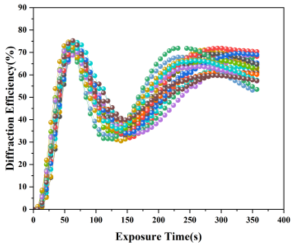

(k)

Figure S4. Diffraction efficiency charts measured with different AIBN for 0.18 g of PQ: (a) 0.15 g; (b) 0.16 g; (c) 0.17 g; (d) 0.18 g; (e) 0.19 g; (f) 0.20 g; (g) 0.21 g; (h) 0.22 g; (i) 0.23 g;(j) 0.24 g;(k) 0.25 g.

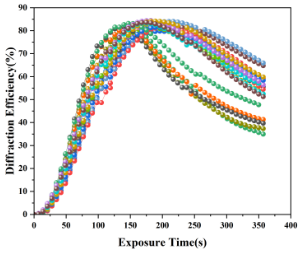

(**a**)

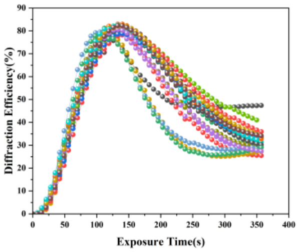

(**b**)

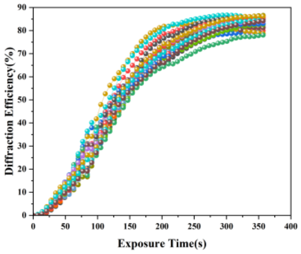

(**c**)

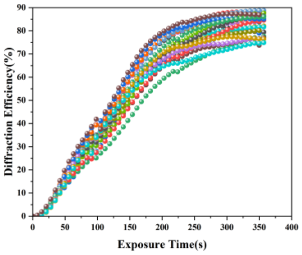

(**d**)

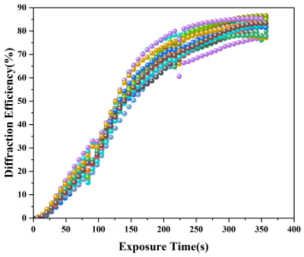

(**e**)

Figure S5. Diffraction efficiency spectra of TPGDA-PQ/PMMA prepared at different temperatures: (a) 68 ℃; (b) 69 ℃; (c) 70 ℃; (d) 71 ℃; (e)72 ℃.

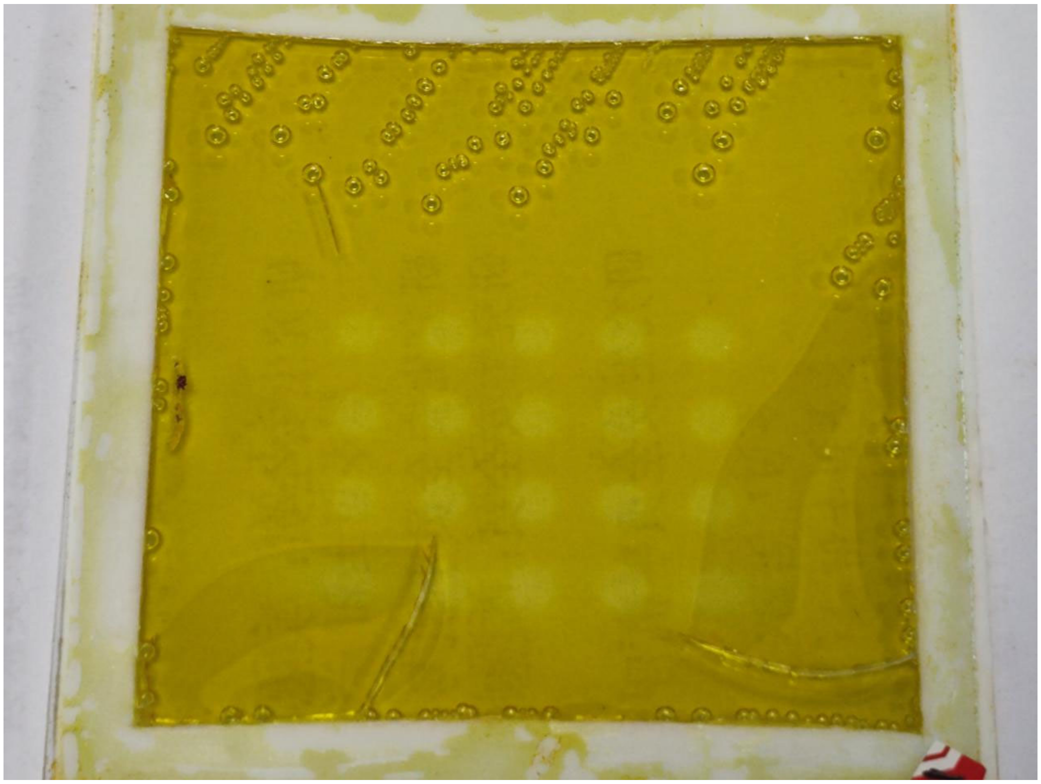

Figure S6. Samples prepared under high-temperature conditions gradually crack after being placed for a period of time.

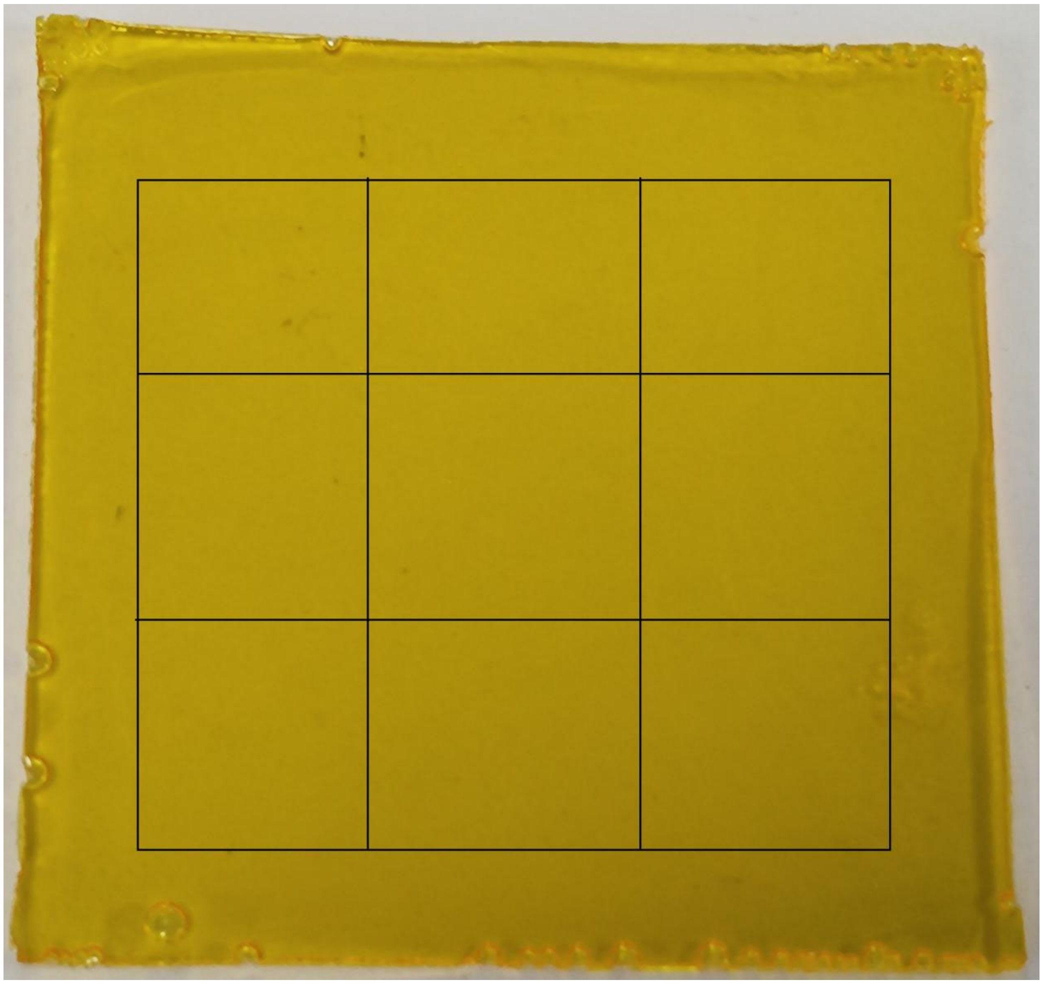

Figure S7. Nine regions for sample division.
